# Children and adolescents with primary headaches exhibit altered sensory profiles – a multi-modal investigation

**DOI:** 10.1186/s10194-024-01819-x

**Published:** 2024-07-09

**Authors:** Michal Pieniak, Berit Höfer, Jenny Knipping, Vanda Faria, Matthias Richter, Valentin A. Schriever, Antje Haehner, Gudrun Gossrau

**Affiliations:** 1 Department of Otorhinolaryngology, Faculty of Medicine Carl Gustav Carus, Smell & Taste Clinic, University Hospital, TU Dresden, Dresden, Germany; 2 Institute of Psychology, University of Wroclaw, Wroclaw, Poland; 3 Interdisciplinary Pain Center, Faculty of Medicine Carl Gustav Carus, University Hospital, TU Dresden, Dresden, Germany; 4 Department of Pediatric Neurology, Faculty of Medicine Carl Gustav Carus, University Hospital, TU Dresden, Dresden, Germany; 5 Department of Pediatrics, Faculty of Medicine Carl Gustav Carus, University Hospital, Dresden, TU Germany; 6 Department of Pediatrics, Pediatric Neurology, Neurometabolics and Prevention, Goethe University Frankfurt, Frankfurt, Germany; 7 Interdisciplinary Pain Center, Faculty of Medicine Carl Gustav Carus, University Hospital, TU Dresden, Fetscherstr. 74, 01307 Dresden, Germany

**Keywords:** Migraine, Tension-type headache, Quantitative sensory testing, Transcutaneous electrical nerve stimulation, Olfaction, Trigeminal

## Abstract

**Background:**

Pediatric headache is an increasing medical problem that has adverse effects on children’s quality of life, academic performance, and social functioning. Children with primary headaches exhibit enhanced sensory sensitivity compared to their healthy peers. However, comprehensive investigations including multimodal sensory sensitivity assessment are lacking. This study aimed to compare sensory sensitivity of children with primary headaches with their healthy peers across multiple sensory domains.

**Methods:**

The study included 172 participants aged 6 to 17 years (M = 13.09, SD = 3.02 years; 120 girls). Of these 80 participants were patients with migraine, 23 were patients with tension-type headache, and 69 were healthy controls. The following sensory measures were obtained: Mechanical Detection Threshold (MDT), Mechanical Pain Threshold (MPT), Mechanical Pain Sensitivity (MPS), detection and pain threshold for Transcutaneous Electrical Nerve Stimulation (TENS), olfactory and intranasal trigeminal detection threshold, and odor identification ability. Sensory sensitivity was compared between groups with a series of Kruskal-Wallis tests. Binomial regression models were used to compare the relative utility of sensory sensitivity measures in classifying participants into patients and healthy controls, as well as into patients with migraine and tension-type headache.

**Results:**

Patients with migraine had lower MPT measured at the forearm than patients with tension-type headaches and healthy controls. MPS was higher in patients with migraine than in healthy controls. All patients with headaches had lower detection threshold of TENS and higher olfactory sensitivity. Healthy controls showed increased intranasal trigeminal sensitivity. Scores in MPS, TENS, and olfactory and trigeminal thresholds were significantly predicting presence of primary headaches. Additionally, scores in MPT, olfactory and trigeminal threshold were positive predictors of type of headache.

**Conclusions:**

Children with primary headaches exhibit different sensory profiles than healthy controls. The obtained results suggest presence of increased overall, multimodal sensitivity in children with primary headaches, what may negatively impact daily functioning and contribute to further pain chronification.

**Trial registration:**

The study was registered in the German Registry of Clinical Trials (DRKS) DRKS00021062.

## Background

Primary headaches (i.e., headaches that are not caused by other diseases or medical conditions [1]) are an increasing medical concern in the pediatric population. The number of children and adolescents experiencing primary headaches has been rising in the last years [2]. A recent meta-analysis concluded that worldwide 11% of children and adolescents experience migraine (8% without aura, 3% with aura), while 17% suffer from tension-type headaches [3]. Pediatric primary headaches are related to increased symptoms of depression and anxiety [4], negatively affect academic performance [5, 6], and may lead to school absence [7], withdrawal from social activities, and compromised well-being [8]. Considering all their negative consequences and high prevalence, pediatric primary headaches constitute one of the major causes of disability in childhood [9].

Patients with primary headaches process sensory stimuli differently than their healthy counterparts [10]. Compared with healthy controls, adult patients with primary headaches have lower thresholds for pressure and heat pain, and perceive suprathreshold cold and electrical stimulation as more painful [11]. Adult migraineurs exhibit increased sensitivity of the intranasal trigeminal system which is reflected in both psychophysical testing and event-related potentials [12, 13]. Moreover, adults with primary headaches, particularly those with migraine, display a unique sensory profile in olfaction: they often report increase sensitivity and aversion to smells (osmophobia) [14, 15], yet their objective test scores suggest that their odor sensitivity is lower than that of healthy individuals. [16, 17; but see: 18].

Although the available data are scarce, research conducted in the pediatric population suggests that children with primary headaches also experience abnormal sensory processing of pain evoked by mechanical stimuli or heat [11, 16], i.e., lower mechanical pain threshold [17]. Parents report multiple difficulties of their children with migraine in processing sensory stimuli in daily situations, particularly in the olfactory and gustatory domains [18]. However, psychophysical evaluation of olfactory sensitivity in children with migraine is lacking.

The findings discussed above support the hypotheses that primary headaches arise from increased sensitivity of peripheral sensory neurons that results in decreased detection threshold for various sensory stimuli [10, 19]. However, the available research on primary headaches in the pediatric population lacked a comprehensive assessment of sensory sensitivity across multiple sensory domains within a single research project. Instead, detection and pain thresholds for various sensory stimuli were examined individually. While there are studies focusing on tactile sensitivity in children with primary headaches [11], these studies usually do not assess other sensory modalities, such as olfaction or the intranasal trigeminal system.

Abnormal olfactory sensitivity in adults is hypothesized to contribute to headache chronification [20], therefore, it urges investigation to what extent the altered sensory profile in the olfactory domain contributes to the experienced difficulties in pediatric patients. Furthermore, it becomes crucial to understand the differences between the olfactory and intranasal trigeminal thresholds of children with primary headaches in contrast to those of healthy children.

The degree of sensory processing difficulties is linked to children’s quality of life [18]. Therefore, understanding the abnormal sensory sensitivity in children with primary headaches is of great importance. In this study, we present a comprehensive, multi-modal comparison of sensory sensitivity in children with two types of primary headaches (migraine or tension-type) and healthy controls. We measured detection and pain thresholds for mechanical and electrical stimuli, and also assessed olfactory and intranasal trigeminal thresholds as well as odor identification ability. Based on the available data from children and adults, we hypothesized that children with primary headaches would exhibit decreased thresholds for intranasal trigeminal stimulation [13] as well as for tactile and electrical stimuli [11], and increased thresholds for odors [21]. Additionally, we evaluated the effectiveness of these sensory measures in distinguishing between healthy children vs. patients, and between children with migraine vs. children with tension-type headache.

## Methods

### Study population

We recruited pediatric patients aged between 6 and 17 years from a tertiary outpatient pain center at a university hospital. The inclusion criteria for patients were (1) a diagnosis of primary headache disorder according to the International Classification of Headache Disorders ICHD IIIb [22] and (2) willingness to participate in the study. Age- and gender-matched healthy participants were recruited using leaflets distributed in schools, local sport clubs, and parental groups as well as the hospital campus. Inclusion criteria for healthy controls included (1) lack of recurring headaches that would affect child’s functioning, (2) lack of other chronic pain disorders, or (3) lack of other neurological disorders.

### Methods

#### Pediatric migraine disability assessment (PedMIDAS)

The severity of disability caused by headaches was assessed with the Pediatric Migraine Disability Assessment (PedMIDAS) scale. PedMIDAS measures the frequency and intensity of headaches and quantifies how often school functioning or other areas of functioning were negatively affected by headaches [23].

#### Quantitative sensory testing (QST)

QST is a standardized psychophysical test battery, measuring somatosensory sensitivity as described elsewhere [24]. We used three components of the test battery, namely Mechanical Detection Threshold (MDT), Mechanical Pain Threshold (MPT), and Mechanical Pain Sensitivity (PinPrick stimulator, von Frey filaments, Optihair2, MRC Systems GmbH, Heidelberg, Germany). Participants were tested according to the published testing protocol [24]. All tests were applied in two locations: control area on the volar forearm and the test area on the cheek, corresponding to the trigeminal nerve V2 branch. High MDT and MPT scores indicate greater thresholds (lower sensitivity), whereas a high MPS score indicates greater pain sensitivity towards the presented stimuli.

#### Transcutaneous electrical nerve stimulation (TENS)

TENS allows to measure sensory sensitivity towards stimuli presented as electric currents applied to the skin through electrodes [25]. Currents are applied with increasing intensity until participants detect the stimulation (detection threshold) and declare that the stimulation evokes pain (pain threshold). The testing area was the volar forearm and the thresholds were obtained for both the left and right limbs. The current was generated using the Omnitens (Neurotech, Ehringshausen, Germany) device. Higher scores in TENS indicate a greater threshold (lower sensitivity) towards the electric stimulation. While pretesting the study procedure, we verified the possibility of additional TENS stimulation in the face area. However, children included in the pretesting sessions reported significant discomfort and TENS stimulation in the face area was not included in the final study protocol.

#### Chemosensory function

All the stimuli used for chemosensory testing were delivered in felt-tip pens filled with odors (Sniffin’ Sticks, Burghart Messtechnik GmbH, Holm, Germany) that are routinely employed in the otorhinolaryngological practice. Olfactory sensitivity was measured with Threshold subtest from the Sniffin’ Sticks battery [26]. In the test, participants are repeatedly presented with triplets of pens, one containing an odor (phenyl ethyl alcohol, PEA) and two containing odorless propylene glycol (PG). During each trial, participants are asked to indicate which pen contains the odor. PEA is prepared in 16 concentrations, starting from a 4% solution in PG that is further diluted in PG in a 1:2 ratio until all concentrations are obtained. Trials start from lowest to highest concentration until participants respond correctly twice in a row at the same concentration level. After two correct responses, participants are presented with lower concentrations until the first incorrect response. This reversed staircase method is employed until seven reversal points are obtained. The final score is the mean of the last 4 reversal points and ranges from 1 to 16 with higher scores indicating greater olfactory sensitivity.

An analogous threshold test with 10 concentration levels was employed to measure intranasal trigeminal sensitivity, with the highest concentration being 50%. The target odor was eucalyptol which is known to evoke both olfactory and trigeminal sensations [27].

Odor identification ability was measured with two odor identification tests – Identification subtest from the Sniffin’ Sticks battery [26] and the U-Sniff test [28]. These tests have 16 and 12 items, respectively. Two tests have been employed as they differ in difficulty (U-Sniff is designed specifically for young children; Sniffin’ Sticks Identification test is used in all age groups) and the age range in the tested group was high. In both tests, participants are presented with an odor (e.g., apple, butter, grass) and a set of 4 possible answers presented in pictures and labeled (1 response is correct, 3 responses are incorrect). Each correct identification is scored with 1 point. Scores from both tests have been summed up and ranged from 0 to 28.

### Procedure

All participants were interviewed about their headache symptoms and filled in the PedMIDAS questionnaire. Further, all the sensory tests have been conducted, starting with QST tests, followed by TENS and chemosensory testing. Sensory sensitivity in patients with primary headaches changes pre-, post- and during the ictal phase [29, 30]. Therefore, patients were tested at least 48 h since the last headache, and were followed up by phone after the study, to verify if they did not experience headache in the next 48 h after the study.

### Statistical approach

All the analyses have been conducted using *R* [31] with a significance level set to *α* = 0.05. We employed packages pROC and pgirmess [32, 33] for data analysis and ggplot2 and cowplot for data visualization [34, 35]. For all the tested variables we calculated the descriptive statistics and assessed normality of distribution using skewness ratio [36]. As the data were not-normally distributed, we used non-parametric analyses. We used the χ2 test of association to verify if groups were balanced in terms of gender distribution. Further, we created a correlation matrix using Spearman correlation. We used a series of Kruskal-Wallis tests to verify differences between patients with migraine, patients with tension-type headache, and healthy controls. For post-hoc comparisons, we used Wilcoxon rank sum test with Bonferroni correction for multiple comparisons. Further, we used binomial logistic regression models to test the utility of sensory measures in classifying participants into (1) healthy and patients groups, and (2) patients with migraine and tension-type headache. The predictors showing the highest correlation (*r* > .70) [37] have been averaged and included in the model as one variable to avoid collinearity. For each classifying model we first included all the measured variables and these variables that appeared to be significant predictors (*p* < .05) or approached the statistical significance level (*p* < .1) were included in an additional model evaluating the final set of predictors. The predictors that remained significant (*p* < .05) in the final model were further used to calculate sensitivity and specificity of the model in classification task with a threshold for predicted probabilities set to 0.50. To compare model fit, we used Akaike Information Criterion (AIC) values with lower AIC values indicating better model fit. Finally, we calculated Spearman correlation coefficients between the duration of the headaches onset and sensory measures.

## Results

### Participants and descriptive statistics

One-hundred seventy-two participants aged 6 to 17 years (*M* = 13.09, *SD* = 3.02) completed the study procedure. Of these, 80 were patients with migraine (59 girls), 23 were patients with tension-type headache (TTH, 18 girls), and 69 were healthy controls (HC, 43 girls). Regarding the time when headaches started, 6 participants declared the onset within the last few months, another 6 approximately half a year before the study, 14 a year before the study, and 76 participants declared that the headaches started more than one year ago. All three groups were balanced in terms of age (χ^2^_2_ = 4.31, *p* = .116) and gender distribution (χ^2^_2_ = 3.20, *p* = .202). The PedMIDAS scores were significantly higher in migraine and TTH patients than in HC (χ^2^_2_ = 122.82, *p* < .001).

**Table 1:** Descriptive statistics for all the measured variables (*n* = 172)

	Migraine (*n* = 80)					Tension-type headache (*n* = 23)					Healthy controls (*n* = 69)					Skewness (*n* = 172)
	Mean	SD	Median	Min	Max	Mean	SD	Median	Min	Max	Mean	SD	Median	Min	Max	
Age	13.54	2.91	14	6	17	13.22	3.01	14	8	17	12.52	3.09	13	6	17	− 0.55
PedMIDAS	35.12	32.57	28.5	2	180	43.65	36.44	29	4	125	0.64	1.92	0	0	11.5	1.99
QST MDT C	0.19	0.04	0.18	0.18	0.38	0.2	0.04	0.18	0.18	0.29	0.27	0.53	0.18	0.13	4.59	15.56
QST MDT T	0.18	0.01	0.18	0.18	0.28	0.18	0.01	0.18	0.18	0.22	0.18	0	0.18	0.18	0.2	5.63
QST MPT C	19.84	24.69	8	5.66	157.59	36.75	44.19	14.93	6.06	181.02	31.19	42.43	17.15	5.66	294.07	3.81
QST MPT T	21.84	25.81	10.56	5.66	103.97	38.01	47.43	9.85	5.66	168.9	27.94	59.82	13	5.66	388.02	5.1
QST MPS C	0.17	0.06	0.18	0.03	0.26	0.15	0.07	0.15	0.04	0.27	0.12	0.06	0.12	0.01	0.26	− 0.12
QST MPS T	0.17	0.06	0.18	0.02	0.26	0.15	0.07	0.16	0.01	0.28	0.14	0.06	0.14	0.03	0.26	− 0.22
TENS Detection L	3.14	1.21	3	1	7	3.43	0.99	3	2	6	4.78	1.35	5	2	8	0.55
TENS Pain L	10.24	5.19	9	3	32	13.65	8.35	10	4	30	11.87	5.69	10	6	32	1.58
TENS Detection R	2.94	1.06	3	1	5	3.17	1.15	3	1	6	4.51	1.29	4	2	8	0.46
TENS Pain R	10.41	5.61	9	3	28	14.7	10.46	11	4	38	11.35	4.91	10	5	30	1.55
Olfactory threshold	8.53	3.5	9.25	1	15.25	10.04	3.22	11.25	2.25	14.5	6.61	3.48	7.5	1	12.5	− 0.36
Trigeminal threshold	8.62	1.63	9.25	1	10	7.84	2.37	8.5	1	10	9.06	1.22	9.5	4.5	10	-2.48
Odor identification	23.4	2.59	24	11	27	23.26	2.65	24	15	26	23.54	2.36	24	15	28	-1.52

Note. QST – Quantitative Sensory Testing, MDT – Mechanical Detection Threshold, MPT – Mechanical Pain Threshold, MPS – Mechanical Pain Sensitivity, TENS – Transcutaneous Electrical Nerve Stimulation, C – Control area forearm, T – Test area trigeminal V2, L –left side, R – right side, SD – standard deviation

Spearman correlation coefficients for all the variables are presented in Table 2. Due to the high correlation between (1) Mechanical Pain Sensitivity in trigeminal and control areas, (2) TENS detection thresholds on the left and right side, and (3) TENS pain thresholds on the left and right side, these pairs of variables have been averaged for the binomial logistic regression models.

**Table 2:** Spearman correlation coefficients for all the tested variables

	1. PedMIDAS	2. Age	3. MDT C	4. MDT T	5. MPT C	6. MPT T	7. MPS C	8. MPS T	9. TENS Detection L	10. TENS Pain L	11. TENS Detection *R*	12. TENS Pain *R*	13. Olfactory threshold	14. Trigeminal threshold	15. Odor identification
1. PedMIDAS	1														
2. Age	0.18*	1													
3. QST MDT C	0.04	− 0.08	1												
4. QST MDT T	0.09	− 0.1	0.34***	1											
5. QST MPT C	− 0.12	− 0.1	0.03	0	1										
6. QST MPT T	− 0.11	− 0.19*	0.04	0.1	0.58***	1									
7. QST MPS C	0.20**	− 0.21**	− 0.1	0.04	− 0.54***	− 0.32***	1								
8. QST MPS T	0.1	− 0.18*	− 0.04	0.04	− 0.40***	− 0.39***	0.81***	1							
9. TENS Detection L	− 0.46***	− 0.06	0.03	− 0.14	0.04	0.07	− 0.14	− 0.12	1						
10. TENS Pain L	− 0.06	0.11	0.04	− 0.06	0.18*	0.29***	− 0.21**	− 0.27***	0.34***	1					
11. TENS Detection R	− 0.43***	− 0.08	− 0.01	− 0.06	0.15*	0.20**	− 0.21**	− 0.24**	0.76***	0.38***	1				
12. TENS Pain R	− 0.02	0.09	− 0.01	− 0.08	0.14	0.28***	− 0.19*	− 0.30***	0.29***	0.90***	0.39***	1			
13. Olfactory threshold	0.28***	0.21**	− 0.02	0.03	− 0.13	− 0.17*	0.04	0.04	− 0.15	− 0.02	− 0.15*	− 0.02	1		
14. Trigeminal threshold	− 0.18*	0.18*	− 0.18*	− 0.23**	0.05	0.04	− 0.07	− 0.02	0.19*	0.09	0.21**	0.11	0.04	1	
15. Odor identification	0.08	0.41***	− 0.06	− 0.09	0.03	− 0.02	− 0.14	− 0.19*	− 0.03	0.05	− 0.05	0.1	0.08	0.20**	1

Note. QST – Quantitative Sensory Testing, MDT – Mechanical Detection Threshold, MPT – Mechanical Pain Threshold, MPS – Mechanical Pain Sensitivity, TENS – Transcutaneous Electrical Nerve Stimulation, C – Control area forearm, T – Test area trigeminal V2, L – left side, R – right side. * *p* < .05; ** *p* < .01; *** *p* < .001; correlations of *r* ≥ .20 or ≤ − 0.20 were highlighted to increase table’s readability

### Between groups comparisons

Groups did not differ in MDT, either for the control area (χ^2^_2_ = 0.98, *p* = .613) or the trigeminal area (χ^2^_2_ = 4.96, *p* = .084). There was also no difference in MPT for the trigeminal area (χ^2^_2_ = 3.47, *p* = .177), but the groups differed in MPT for the control area (χ^2^_2_ = 11.64, *p* = .003). Specifically, migraine patients had significantly lower MPT thresholds than TTH patients (*p* = .025) or HC (*p* = .011). There was no difference between TTH patients and HC (*p* > .99).

**Fig. 1:**
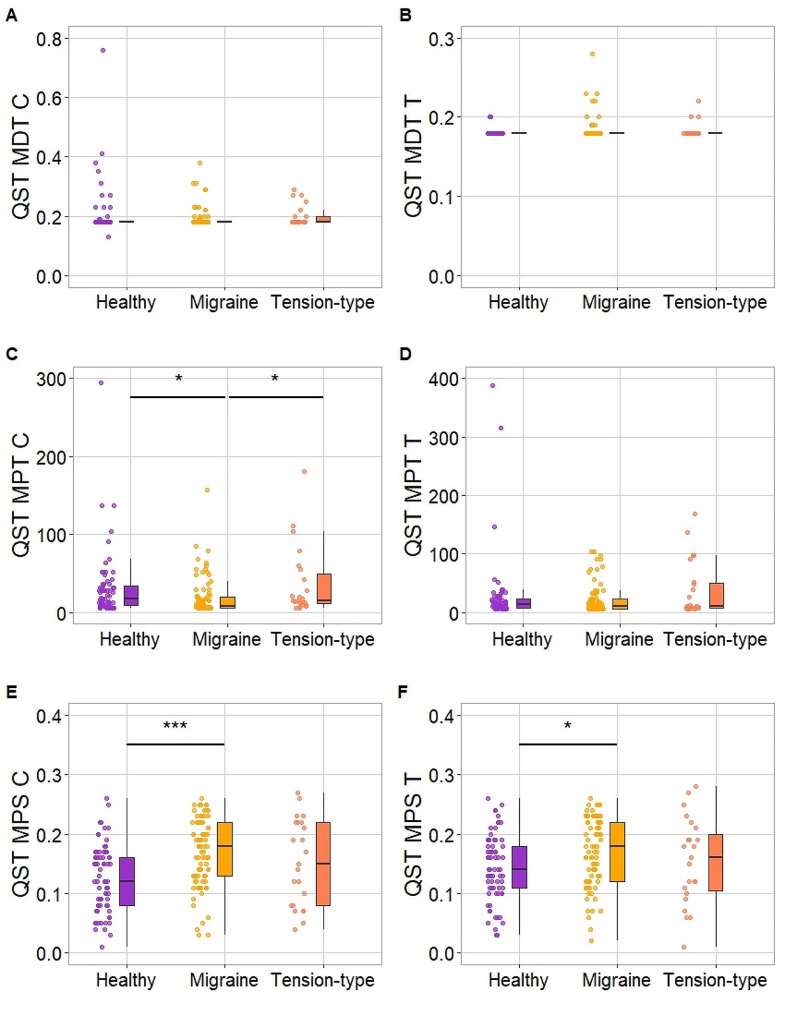
Quantitative Sensory Testing scores between groups. Mechanical Detection Threshold for the control and trigeminal areas is presented in Panels **A** and **B**. Mechanical Pain Threshold for the control and trigeminal areas is presented in Panels **C** and **D**. Mechanical Pain Sensitivity for the control and trigeminal areas is presented in Panels **E** and **F** Note. * - *p* < .05; *** - *p* < .001. C – Control area forearm, T – Test area trigeminal V2. In the Panel A one participant with score 4.59 is not presented in the healthy group to ensure plot’s readability

We found significant differences in MPS in both control and trigeminal areas (χ^2^_2_ = 18.63, *p* < .001; χ^2^_2_ = 8.26, *p* = .016; respectively). In both areas, the sensitivity was increased in migraine patients as compared with HC (*p* < .001, *p* = .011, respectively). TTH patients did not differ from HC or from migraine patients in any of the areas (all *p* > .350). All the results from QST are presented in Fig. 1.

We found significant differences in TENS detection thresholds on both left and right sides (χ^2^_2_ = 51.86, *p* < .001; χ^2^_2_ = 49.15, *p* < .001; respectively). Post hoc analyses showed a similar pattern of results, i.e., both migraine and TTH patients had lower detection thresholds on both sides as compared to HC (all *p* < .001). The patient groups did not differ (*p* = .680, *p* > .99, for the left and right sides respectively).

**Fig. 2:**
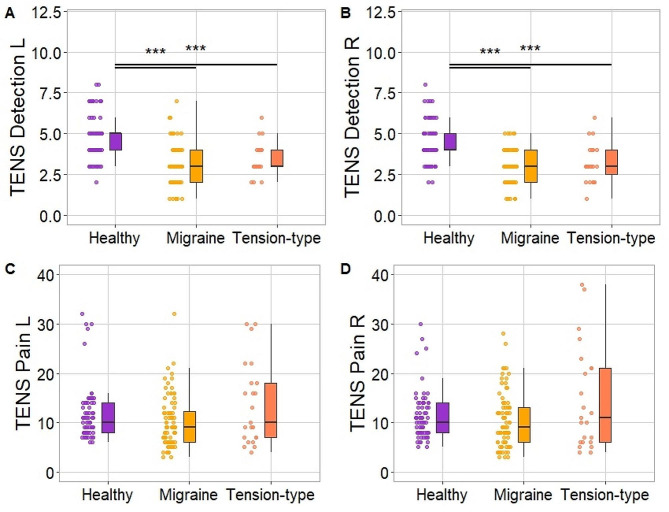
Transcutaneous Electrical Nerve Stimulation scores between groups. Detection thresholds for the left and right sides are presented in Panels **A** and **B**. Pain thresholds for the left and right sides are presented in Panels **C** and **D**. Note. *** - *p* < .001

Groups did not differ in TENS pain thresholds for the left and right sides (χ^2^_2_ = 5.04, *p* = .081; χ^2^_2_ = 3.20, *p* = .202; respectively). TENS scores across groups are presented in Fig. 2.

Patients with migraine and TTH showed higher scores in the olfactory threshold test, indicating increased olfactory sensitivity, χ^2^_2_ = 19.33, *p* < .001. Both groups scored higher than HC, (*p* = .007 for migraine patients, *p* < .001 for TTH patients). Olfactory sensitivity was not different between the two patient groups (*p* = .121).

For the trigeminal threshold, we found an opposite effect, χ^2^_2_ = 10.50, *p* = .005. HC showed greater trigeminal sensitivity as compared to migraine patients (*p* = .036) and TTH patients (*p* = .019). Patients did not differ in the trigeminal sensitivity levels (*p* = .582).

**Fig. 3:**
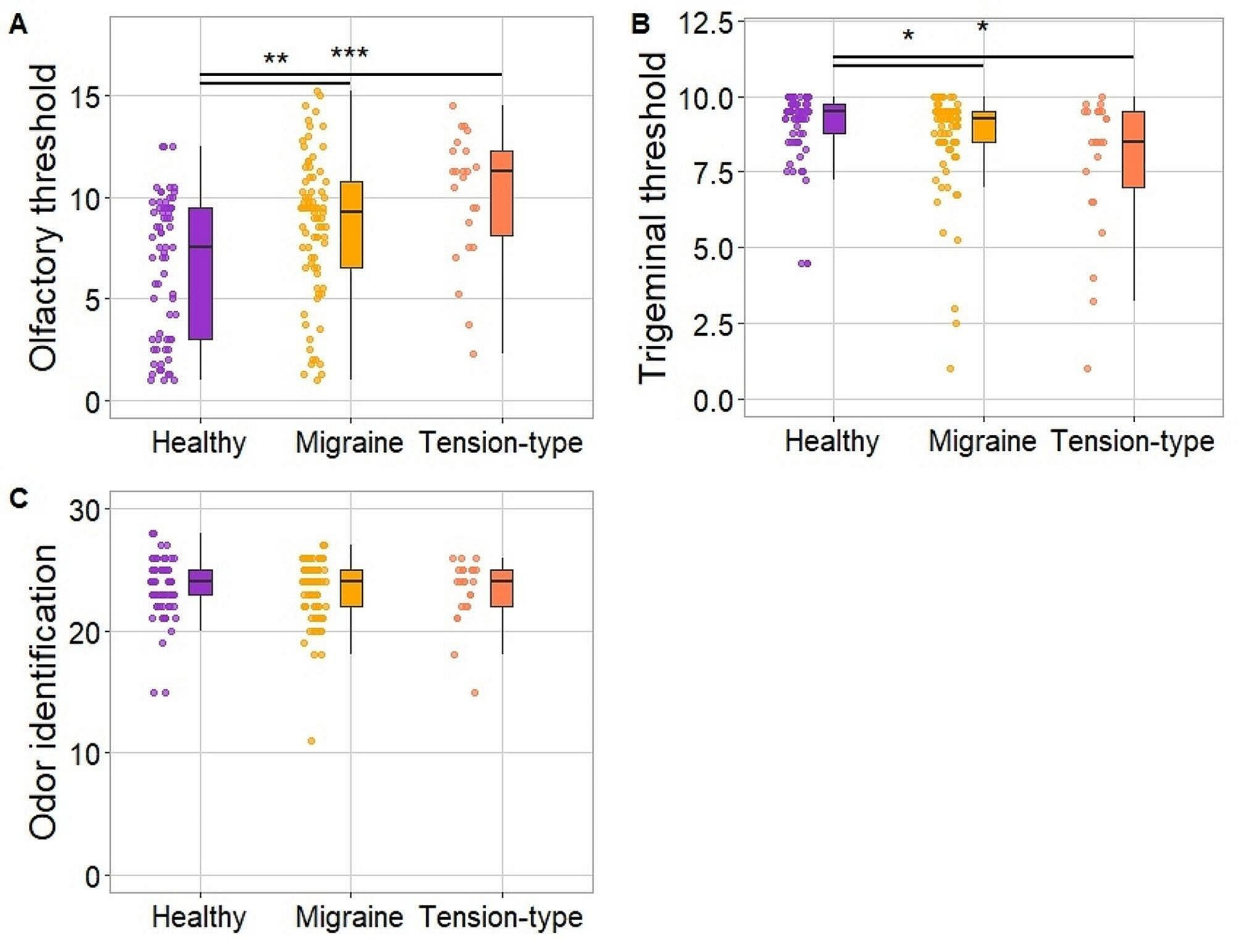
Between-groups comparisons of chemosensory abilities: olfactory threshold (Panel **A**), trigeminal threshold (Panel **B**), and odor identification ability (Panel **C**). Note. * - *p* < .05; ** - *p* < .01; *** - *p* < .001; higher scores in Sniffin’ Sticks threshold tests indicate lower detection thresholds

Groups did not differ in odor identification ability, χ^2^_2_ = 0.02, *p* = .991. All the results related to the chemosensory function are presented in Fig. 3.

### Regression models

The first binomial logistic regression model classifying participants into healthy and patient groups demonstrated that age, MPS, olfactory threshold, trigeminal threshold, and TENS detection and pain threshold are significant or trend-level predictors of the group classification (see Table 3 for regression coefficients and *p*-values). Therefore, all these variables were included as predictors in the final model. The final model showed that MPS (*z* = 3.51, *p* < .001), olfactory threshold (*z* = 2.88, *p* = .004), trigeminal threshold (*z*=-2.07, *p* = .038), TENS detection (*z*=-5.66, *p* < .001) and pain (*z* = 3.00, *p* = .003) thresholds were all significant predictors. These results indicate that variability in these sensory measures aided classification of participants into healthy and patient groups, with higher MPS, olfactory sensitivity, and TENS pain threshold increasing the likelihood of being classified as patient, and higher trigeminal sensitivity and TENS detection threshold decreasing this likelihood. AIC values decreased from 148.6 for the first model to 143.8 for the final model, suggesting a better fit for the latter one. Overall, scores in MPS, olfactory and trigeminal thresholds, TENS detection and pain thresholds classified participants into healthy or patient groups with sensitivity of 87% and specificity of 75%.

**Table 3:** Binomial regression models classifying participants into healthy and patient groups

Predictor	Estimate	SE	z value	*p* value
Model 1				
Intercept	-10.72	11.94	− 0.90	0.369
Age	**0.21**	**0.10**	**2.20**	**0.028**
QST MDT C	-3.11	6.13	− 0.51	0.612
QST MDT T	-79.94	62.02	1.29	0.197
QST MPT C	− 0.002	0.01	− 0.30	0.765
QST MPT T	0.003	0.01	0.52	0.601
QST MPS	**15.14**	**4.96**	**3.05**	**0.002**
Olfactory threshold	**0.19**	**0.07**	**2.76**	**0.006**
Trigeminal threshold	**− 0.44**	**0.23**	**-1.92**	**0.054**
Odor identification	− 0.06	0.12	− 0.48	0.633
TENS Detection threshold	**-1.43**	**0.26**	**-5.44**	**< 0.001**
TENS Pain threshold	**0.13**	**0.05**	**2.74**	**0.006**
Model 2				
Intercept	2.41	2.13	1.12	0.259
Age	0.17	0.09	1.95	0.051
QST MPS	**15.77**	**4.49**	**3.51**	**< 0.001**
Olfactory threshold	**0.19**	**0.07**	**2.88**	**0.004**
Trigeminal threshold	**− 0.44**	**0.21**	**-2.07**	**0.038**
TENS Detection threshold	**-1.47**	**0.26**	**-5.66**	**< 0.001**
TENS Pain threshold	**0.13**	**0.04**	**3.00**	**0.003**

Note. Healthy coded as 0, patients coded as 1

QST – Quantitative Sensory Testing, MDT – Mechanical Detection Threshold, MPT – Mechanical Pain Threshold, MPS – Mechanical Pain Sensitivity, TENS – Transcutaneous Electrical Nerve Stimulation, C – Control area forearm, T – Test area trigeminal V2

The initial model classifying patients into TTH and migraine groups showed that only olfactory and trigeminal thresholds are significant predictors of the classification (*z*=-2.66, *p* = .008; *z* = 2.46, *p* = .014), with MPT for the trigeminal area approaching a significance level (*z*=-1.88, *p* = .060; full model presented in Table 4). These three predictors were the ones included in the final model and all of them became statistically significant. Trigeminal threshold (*z* = 2.48, *p* = .013) was a positive predictor, indicating that higher trigeminal sensitivity increased likelihood of being a migraine patient. Contrary, olfactory threshold (*z*=-2.58, *p* = .010) and MPT for the trigeminal (*z*=-2.73, *p* = .006) area were negative predictors, indicating that greater olfactory sensitivity and higher MPT decreased the likelihood of classification as a migraine patient. AIC values decreased from 110.98 for the initial model to 101.59 for the final model, suggesting a better fit of the latter one. Overall, scores in MPT of the trigeminal area, olfactory threshold, and trigeminal threshold classified patients into migraine and TTH groups with sensitivity of 95% and specificity of 26%. When the classification threshold was adjusted from 0.50 to 0.75, the classifier had sensitivity of 79% and specificity of 57%.

**Table 4:** Binomial regression models classifying patients into migraine and tension-type groups

Predictor	Estimate	SE	z value	*p* value
Model 1				
Intercept	0.77	5.98	0.13	0.896
Age	0.05	0.10	0.45	0.651
QST MDT C	-6.57	7.74	− 0.85	0.396
QST MDT T	14.78	25.40	0.58	0.561
QST MPT C	− 0.01	0.01	-1.26	0.209
QST MPT T	**− 0.02**	**0.01**	**-1.88**	**0.060**
QST MPS	-2.20	5.67	− 0.39	0.698
Olfactory threshold	**− 0.26**	**0.10**	**-2.66**	**0.008**
Trigeminal threshold	**0.36**	**0.10**	**2.46**	**0.014**
Odor identification	0.01	0.15	0.11	0.913
TENS Detection threshold	− 0.25	0.29	− 0.89	0.373
TENS Pain threshold	− 0.04	0.05	− 0.78	0.436
Model 2				
Intercept	1.22	1.23	0.99	0.320
QST MPT T	**− 0.02**	**0.01**	**-2.73**	**0.006**
Olfactory threshold	**− 0.23**	**0.09**	**-2.58**	**0.010**
Trigeminal threshold	**0.33**	**0.14**	**2.48**	**0.013**

Note. Tension-type coded as 0, migraine coded as 1

QST – Quantitative Sensory Testing, MDT – Mechanical Detection Threshold, MPT – Mechanical Pain Threshold, MPS – Mechanical Pain Sensitivity, TENS – Transcutaneous Electrical Nerve Stimulation, C – Control area forearm, T – Test area trigeminal V2

### Correlation between sensory sensitivity and headache duration

The time since the first presence of headaches did not correlate with any of the sensory measures from QST (*r* ≤ .15, *p* ≥ .131), TENS (*r* ≤ .09, *p* ≥ .361) or olfactory tests (*r* ≤ .10, *p* ≥ .315). Only for the trigeminal threshold we found a weak, negative, significant correlation (*r*=-.21, *p* = .31) demonstrating that trigeminal sensitivity was higher in the participants whose headaches started recently and decreased in patients who experienced symptoms for several years.

## Discussion

The presented study aimed to compare detection and pain threshold across multiple sensory domains between children with primary headaches (migraine and tension-type) and their healthy peers. We demonstrated that, regardless of headache type, children with primary headaches had similar detection thresholds for mechanical stimuli as healthy controls. Mechanical pain thresholds on the volar forearm were lower in migraineurs as compared with healthy controls and tension-type headache patients. Migraineurs also reported increased subjective sensitivity to suprathreshold mechanical stimulation. These results are in line with studies in the adult population pinpointing increased sensitivity to painful tactile stimuli in migraine despite having typical detection thresholds for mechanical stimulation [11].

Further, we observed that children with primary headaches had lower detection thresholds for electrical stimulation, although pain thresholds for electrical stimulation did not differ between patients and the healthy group. This finding is in contrast with reports from the adult population where no differences in detection and pain thresholds for electrical stimulation are found between patients with primary headaches and healthy controls [11]. It suggests that children with primary headaches detect electrical stimulation at lower amperage but this increased sensitivity towards electrical stimuli diminishes over time. However, longitudinal studies are required to verify this notion.

Interestingly, we found that the olfactory sensitivity is higher in children with primary headaches than in healthy controls. This contributes to the body of data demonstrating overall increased sensitivity in different sensory domains. This finding is even more intriguing considering that the available research suggests decreased olfactory sensitivity in adult migraineurs [21]. There is however a possible trajectory of olfactory sensitivity decline in migraineurs from childhood to adulthood, related to exposure to odors. Taking into account that adolescents with migraine are less likely to seek sensory input than their healthy peers [38], it is plausible that children who are overly sensitive to smells try to avoid olfactory inputs and spend time in odor-free environments. Such avoidance might lead to an impaired olfactory threshold in adulthood as research shows that a large number of odors spotted in one’s environment boost olfactory abilities [39] whereas functioning in odorless conditions diminishes olfactory sensitivity [40, 41]. This hypothesis requires verification in longitudinal studies that would follow changes in the olfactory thresholds of individuals with primary headache from childhood to adulthood. Such research would help to understand the role of olfactory sensitivity and osmophobia in the development and chronification of headache [20].

Additionally, a better understanding of the contributions of olfactory sensitivity to chronic headaches may lead to more efficient use of systematic exposure to odors, i.e., olfactory training, in mitigating headaches. To date, olfactory training has been shown to increase pain thresholds in adult patients with chronic lower-back pain [42] and in children with primary headaches [43]. Studies in patients with olfactory dysfunction demonstrate that olfactory training leads to structural and functional changes in multiple brain areas [44], including increased functional connectivity within regions involved in pain processing [45]. Whether such functional reorganization of pain networks occurs in migraineurs following olfactory training and how it relates to the experienced symptoms is yet to be verified [46].

Odor identification ability did not differ between the study groups. The lack of differences can be expected considering that identifying odors relies more on semantic knowledge and does not only depend on peripheral sensitivity of the olfactory system [47]. Odor identification tests are most commonly used in research and clinical practice due to their simplicity and time efficiency [48]. However, our results suggest that when examining olfactory function in patients with headaches, olfactory threshold tests should be preferred as they are more closely related to the experienced symptoms and may be more informative for researchers and clinicians.

Finally, children with primary headaches had increased intranasal trigeminal thresholds which is contrary to findings reported in adults [12, 13]. We speculate that the opposite direction of the effect observed in our study might arise from the assessment method. Firstly, we used eucalyptol, as the target stimulus, which is a bimodal stimulus evoking both trigeminal and olfactory sensations. Secondly, trigeminal stimuli evoke different sensations (e.g., cooling, burning, tingling) and eucalyptol used in the threshold test evokes cooling sensations that might be less related to pain processing than more aversive trigeminal stimuli like capsaicin. Finally, the distribution of the scores suggests the presence of a ceiling effect (most of the participants had very low thresholds) which interferes with an accurate comparison of thresholds across groups. For these reasons, future studies could use different methods of intranasal trigeminal function assessment such as lateralization test [49], automated carbon dioxide threshold measurement [50] or trigeminal event-related potentials [51].

We did not find a correlation between disease duration and sensory sensitivity (with the exception of intranasal trigeminal sensitivity). This is in contrast to previous studies in adults that demonstrated that patients with an earlier onset of migraine showed increased neural responses to trigeminal stimulation than patients whose symptoms emerged more recently [13, 52]. However, the lack of correlation in the presented study might be due to the different age of patients with migraine: we investigated children and adolescents, whereas former studies reported data on sensitization in adult migraineurs. In addition, our results can be statistically con-founded by homogeneity of the study sample (almost 75% of participants had migraines for more than a year). Limited variability of the disease duration within a study sample hinders the ability to detect linear relationships between the duration and sensory measures.

Our findings suggest unique sensitivity in children with migraine and TTH. The observed hypersensitivity to various sensory stimuli might arise from hyperreactivity of peripheral sensory neurons [10], impaired modulation and gating of sensory inputs by brain stem and hypothalamic nuclei [19, 53], dysfunctional neuronal filtering of sensory input by pain inhibitory circuits in the prefrontal cortex and rostral anterior cingulate cortex [54] or overall cortical disinhibition and impaired habituation [30, 55–57]. In adult migraineurs, altered cerebral filtering of incoming stimuli has been reported not only for tactile and olfactory stimuli, but also for negative emotional facial expressions, pinpointing to widespread impairment of aversive stimuli processing [58]. Comparative analyses of sensory measures show that subjective sensitivity to mechanical pain and detection thresholds for electric, olfactory, and intranasal trigeminal stimuli distinguish healthy individuals and patients with primary headaches. Additionally, the determination of olfactory and trigeminal thresholds further helps to classify patients with headaches into migraine and tension-type groups.

The sensory hypersensitivity observed in children with primary headaches might impair their efficient functioning and limit social activities in sensory-disturbing environments. For instance, noisy and odorous school environment might hinder academic performance, decrease the motivation to participate in extracurricular programs, and engage in social activities. To some extent, these issues might be counterbalanced with therapy. Available interdisciplinary therapy programs aim to support patients, for instance, by teaching them mindfulness and relaxation techniques, educating on potential stressors triggering headaches [59, 60]. Complementary institutional support might be additionally considered, such as providing a possibility of taking school exams in classrooms with limited sensory inputs.

The sample size of the study did not allow us to run sub-group analyses focusing on chemosensory functions in migraineurs with and without aura, whereas in the adult populations, these groups differ in chemosensory processing [61]. This constitutes a limitation of the presented research. Additionally, we included relatively few patients with tension-type headaches which limits generalizability of our findings regarding this population. Another limitations of the study is lack of thermal sensitivity assessment. Previous research examining sensory processing in children and adults with migraine demonstrated increases sensitivity towards thermal stimulation [11], but we did not include this measure in the study protocol to avoid making the procedure too lengthy and demanding for children. Therefore, contribution of the thermal sensitivity to the effects observed in our study awaits further verification. Finally, some of the measures used in the study (such as the PedMIDAS scale) are self-reports that are prone to recall bias.

Future studies would greatly benefit from including neuroimaging methods. Such an approach would allow uncovering mechanisms driving sensory alterations in patients with primary headaches. Longitudinal research is necessary to better understand the trajectory of developmental changes in sensory sensitivity across different modalities in patients with primary headaches. Another avenue of research is verification if the negative impact of sensory hypersensitivity on children’s social and academic functioning might be mitigated by creating less sensory-disturbing environments at homes and schools.

## Conclusions

Children with primary headaches exhibit increased sensitivity towards multiple sensory stimuli, including odors, electrical and mechanical stimulation. These differences in sensory sensitivity aid classification into healthy vs. headache, and migraine vs. tension-type groups. The increased sensing of diverse sensory stimuli in children with primary headaches may hinder their academic and social functioning. Targeted behavioral interventions and supportive measures might mitigate the negative impact of sensory hypersensitivity on daily lives of children with primary headaches.

## Data Availability

No datasets were generated or analysed during the current study.

## Abbreviations

HC: healthy controls
MDT: Mechanical Detection Threshold
MPS: Mechanical Pain Sensitivity
MPT: Mechanical Pain Threshold
TENS: Transcutaneous Electrical Nerve Stimulation
TTH: tension–type headache
QST: Quantitative Sensory Testing
